# Scaling a Brief Digital Well-Being Intervention (the Big Joy Project) and Sociodemographic Moderators: Single-Group Pre-Post Study

**DOI:** 10.2196/72053

**Published:** 2025-06-04

**Authors:** Darwin A Guevarra, Yoobin Park, Xuhai Xu, Jin Liou, Jolene Smith, Peggy Callahan, Emiliana Simon-Thomas, Elissa S Epel

**Affiliations:** 1 Department of Psychology Miami University Oxford, OH United States; 2 Department of Psychiatry and Behavioral Sciences University of California, San Francisco San Francisco, CA United States; 3 Department of Biomedical Informatics Columbia University New York United States; 4 Mission: JOY, the JOY Film LLC Los Angeles, CA United States; 5 Greater Good Science Center University of California, Berkeley Berkeley United States

**Keywords:** well-being, internet-based intervention, digital health promotion, socioeconomic factors, subjective social status, low-cost intervention, mental health, public health

## Abstract

**Background:**

Emotional well-being interventions lead to better mental and physical health. However, most of these interventions have been tested on relatively homogeneous samples, with few interventions large enough to examine whether key sociodemographic factors impact outcomes. In addition, barriers to engagement include access and high participant burden. We developed a brief web-based intervention to address these barriers and tested the effects across sociodemographic groups.

**Objective:**

The study aims to examine the effectiveness of a brief, low-burden digital well-being intervention in improving emotional well-being and health-related outcomes across a diverse global sample. It investigates how key sociodemographic factors, such as age, sex, race and ethnicity, education, financial strain, and subjective social status, moderate intervention effects. The goal is to identify which groups benefit most, informing the scalability and public health potential of digital well-being interventions.

**Methods:**

We conducted a single-group pre-post study spanning from 2022 to 2024 using a web-based, multicomponent, week-long well-being intervention requiring 5 to 10 minutes of daily activity (the Big Joy Project). Using a global convenience sample recruited via open web-based enrollment, we assessed pre-post changes in emotional well-being, positive emotions, happiness agency, perceived stress, self-reported health, and sleep quality. At baseline, participants also reported sociodemographic characteristics. We used mixed-effects linear models to examine pre-post changes in the outcomes and sociodemographic moderators.

**Results:**

The sample (N=17,598) consisted of individuals from 169 countries and territories, with broad representation across sociodemographic groups; however, the sample was predominantly White, female, and had at least a high school or college education. Following the intervention, participants showed significant within-subjects effect size increases in emotional well-being (*d_z_*=0.48; *P<*.001), positive emotions (*d_z_*=0.45; *P<*.001), and happiness agency (*d_z_*=0.44; *P<*.001). Furthermore, participants showed a decrease in perceived stress (*d_z_*=–0.35; *P<*.001) and an increase in self-reported health (*d_z_*=0.07; *P<*.001) and sleep quality (*d_z_*=0.15; *P<*.001). There was a clear dose-response pattern across outcomes: participants who engaged in more daily practices showed greater improvements. There was a strong pattern of social disadvantage moderating these effects, with groups experiencing greater social disadvantage showing larger benefits across most outcomes. For example, those with lower education, greater financial strain, or lower subjective social status and those identifying as individuals from racial or ethnic minority groups (Black or Hispanic) all showed larger improvements across well-being outcomes. Furthermore, younger people had greater increases in emotional well-being and greater decreases in perceived stress compared to older people.

**Conclusions:**

A brief, low-intensity intervention showed meaningful improvements in well-being and stress, comparable to those seen in longer, more intensive digital well-being interventions. Sociodemographic groups that are at higher risk of poor mental health benefited more from the intervention, highlighting its potential for scalable public health impact. Testing this intervention with a randomized controlled trial design will be important.

## Introduction

### Background

The US National Prevention, Health Promotion, and Public Health Council identified mental and emotional well-being as 1 of 7 priority areas in a National Prevention Strategy to improve public health [1]. There is growing evidence that improvements in well-being are associated with a decreased risk of future mental illness [2,3] and better physical health [4,5]. Consequently, Feller et al [6] from the Los Angeles Community Translational Science Team proposed a national emotional well-being initiative to improve public health [7]. In parallel, there is a proliferation of research on interventions to improve emotional well-being [8]. Although these interventions are largely effective, they often require time, personal resources, and ongoing effort, which are barriers to large-scale adoption [9]. Moreover, the effects of these interventions are heterogeneous, and large-scale studies are needed to identify key sociodemographic variables that may moderate their effects [8,10]. In this paper, we test a brief, web-based well-being intervention, the Big Joy Project, on a large global sample to examine the impact of sociodemographic factors.

### Emotional Well-Being and Mental and Physical Health

Emotional well-being is a multidimensional composite that includes both the quality of momentary experiences and broader life judgments [7]. Emotional well-being is intimately associated with mental and physical health [4,5,11,12]. For example, people with lower emotional well-being are at a greater risk of being diagnosed with depression at a later time [2,13,14]; conversely, people with higher emotional well-being have reduced incidence of both future anxiety and depression [15,16] and lower long-term risk of suicide [17]. Emotional well-being also predicts better physical health. Higher emotional well-being is associated with a lower risk of developing different chronic conditions, such as cardiovascular diseases [18], arthritis [19,20], and chronic lung disease [21]. People with higher emotional well-being also report less physical decline, more physical activity, longevity, and reduced mortality in healthy and unhealthy populations [4,22-24]. Given these protective effects, it is not surprising that there is great interest among health policy leaders and the public in promoting emotional well-being to improve public mental and physical health.

### Emotional Well-Being Interventions

Because various dimensions of emotional well-being are related to health [25], researchers have suggested interventions for promoting it as a strategy to improve health in both the general population and those with medical conditions [26]. These interventions can be single or multicomponent. Single-component intervention studies typically use 1 to 2 positive psychology activities that target 1 to 2 facets of well-being, such as studies on the effects of gratitude (eg, feeling grateful, gratitude journal, gratitude letter, and gratitude expression) [27], acts of kindness [28,29], and reflecting upon or aligning daily activities with character strengths [30]. Multicomponent interventions contain several positive psychology activities and target ≥2 facets of emotional well-being within a typically more extensive program. A recent quantitative synthesis of meta-analytic evidence reported that positive psychology interventions significantly increase emotional well-being [31].

Multicomponent emotional well-being interventions tend to be more effective than single-component ones [25]. For example, Heintzelman et al [32] tested a 12-week comprehensive intervention program, Enduring Happiness and Continued Self-Enhancement (ENHANCE), through in-person or web-based sessions totaling 36 to 48 hours. Both formats of ENHANCE, compared to a waitlist control group, increased well-being (*d*=0.30) and positive affect (*d*=0.46) and decreased stress (*d*=–0.55). Martin [33] tested an abridged version of ENHANCE (5 weeks instead of 12 weeks) using a pre-post design and found smaller effects on well-being (*d_z_*=0.37 vs to *d_z_*=0.67), suggesting that longer interventions may lead to larger effects [32,33].

To scale well-being interventions, Prydz et al [34] tested a 10-week web-based version of the Five Ways to Wellbeing for All (5waysA) program. The first 5 weeks included a 2-hour webinar and a 1-hour booster webinar to educate people about how stressors in life impact well-being and health and how the program can help reduce stress, increase positive emotions, and improve well-being. The following 5 weeks featured twice-weekly text reminders with practice suggestions. Similar to ENHANCE, 5waysA, compared to the control group, increased well-being (*d*=0.30) and positive affect (*d*=0.49). Although the authors did not provide within-person effect sizes, we estimate that, from baseline to postintervention (assuming a correlation of 0.50), the program increased within-person well-being by approximately *d_z_*=0.35 and positive affect by *d_z_*=0.52.

Although lengthier well-being interventions are important, such as ENHANCE [32] and 5waysA [34], many people may lack the time, motivation, and resources to engage in more extensive well-being intervention programs [33]. While web-based interventions are becoming increasingly popular to scale these programs, they often have high attrition rates [35-37]. One of the key barriers to people engaging in emotional well-being interventions is that they are more time intensive and require ongoing effort [38,39]. Therefore, it is also important to develop less time-intensive and low-effort programs and test if they can lead to meaningful changes in emotional well-being and health-related outcomes.

### Key Moderators of Well-Being Interventions

Kubzansky et al [8] point out that although well-being interventions are generally effective, their impact varies across people. Understanding the source of this heterogeneity is key to understanding which interventions work best for whom [10]. For example, sociodemographic factors, such as age, sex, and race and ethnicity, may moderate intervention effects. Traditional meta-analyses are limited in assessing these individual differences [40,41]. This may help explain the mixed findings on how key sociodemographic factors moderate the effects of well-being interventions. For example, Carr et al [25] found that older people benefited more than younger people, whereas previous meta-analyses found no moderator effect of age [42]. Socioeconomic status may also play a role. One of the criticisms of the positive psychology literature and well-being interventions is that they typically target people with more privilege, such as those who are more well educated and wealthier [43]. Moreover, those who are older, White, and have less financial strain are more likely to initiate and adhere to well-being interventions [37]. Larger and more diverse studies are needed to explore how well-being interventions perform across subgroups.

### Study Overview

We conducted a global, single-arm, pre-post web-based intervention study for 7 days, the Big Joy Project. The Big Joy Project is a citizen science initiative designed to advance the science of well-being and improve public health. It was developed to share and expand the science of positive well-being, an underlying theme for the documentary *Mission: JOY—Finding Happiness in Troubled Times*. Our study had 2 primary objectives. First, we examined whether this brief web-based intervention program (approximately 5-10 min daily for 7 days) could enhance well-being, decrease stress, and improve health. Specifically, we explored multiple outcomes, including emotional well-being, positive emotions, agency over one’s happiness, perceived stress, self-reported health, and sleep quality. All outcomes were assessed at baseline and at the end of the 7-day program. We also tested if the number of activities completed moderated these main effects. Second, we tested whether 6 sociodemographic variables moderated the impact of the Big Joy program on our primary outcomes: age, sex, race and ethnicity, education, subjective social status, and financial strain.

## Methods

### Procedure

The Big Joy Project invited people to join a 7-day program that involved engaging daily in a brief microact of joy (takes approximately 5-10 min daily). We described the study to people as a way to explore which microact of joy might work for them to minimize the effects of positive expectations. Participants were recruited via a global convenience sampling strategy using open enrollment through a web link following in-person and web-based film screenings, email newsletters from *Mission: JOY* and partner organizations (eg, Mind and Life Institute), social media postings (eg, Facebook [Meta Platforms, Inc] and Instagram [Meta Platforms, Inc]), academic conferences, media interviews about the film, website content (eg, promotional articles and images), and word of mouth. The project was funded by the *Mission: JOY* organization, individual donors, and foundations. For this study, the data were collected from June 1, 2022, to February 1, 2024. Inclusion criteria were minimal and included being aged at least 18 years and providing informed consent. There were no exclusion criteria because the study aimed to assess intervention outcomes in a broad, real-world population.

People interested in participating were directed to a website and asked to complete 1 activity or microact. Before the microact, participants reported their current positive and negative feelings, listened to a brief audio clip of different people laughing, and then reported their positive and negative feelings again. This was followed by 2 questions on a slider scale related to the ease (how easy or difficult was that act of joy for you?—very difficult to very easy) and fit of the practice (did that act of joy feel like a good fit, like you would want to do it again at another time?—not at all to completely).

After the example activity, people provided informed consent and completed an onboarding survey that included sociodemographic and psychological measures. For the next 7 days, participants received an email in the morning (8 AM local time) prompting them to engage in a different, randomly ordered activity. At the end of 7 days, people completed the same psychological measures they saw on the onboarding survey.

### Ethical Considerations

This study was approved by the institutional review board at the University of California, Berkeley (protocol 2021-01-13936), and all procedures followed institutional ethical guidelines for research involving human participants. Informed consent was obtained electronically from all participants before participation, and they were informed of their right to withdraw at any time without penalty. Data were collected anonymously, and all data were deidentified during analysis to ensure participant privacy and confidentiality. Participants were not offered any form of compensation for their participation in the study.

### Interventions

Participants were prompted to engage in a daily brief activity for 7 days. Before and immediately after each activity, participants were asked how much they felt pleasant emotions such as delight, pride, and hope and unpleasant emotions such as distress, sadness, and anger. After each activity, they were also asked how difficult the activity was and if they thought it was a good fit for them. Each activity was designed to be brief and took between 5 and 10 minutes. For all activities, we operationalized and estimated the duration of the prompt and how long it took to finish it. Some activities could only be completed offline and required interaction with other people (eg, celebrating another’s joy or doing something kind). For these activities, we did not include that additional time in our duration estimate. The order of activities sent to each participant was randomized. The 7 activities in the Big Joy Project are as follows:

- Celebrate another’s joy. This prompted people to find someone to talk to and asked them to share a fun, inspiring, or proud moment. (Takes approximately 5 min)
- Shift your perspective. This prompted people to think and write of a recent time when they felt frustrated, upset, or anxious and then write 3 positive things that came out of it. (Takes approximately 8 min)
- Do something kind. This prompted people to think of up to 5 individuals they might see that day and list 1 thing that they could do to brighten the person’s day. (Takes approximately 10 min)
- Tune in to what matters. This prompted people to rank 4 values (virtue, fairness, goodwill, and unity) in order of importance and then write about how the values appear in their life. (Takes approximately 10 min)
- Make a gratitude list. This prompted people to think, reflect, and list up to 8 things or people they feel grateful for. (Takes approximately 10 min)
- Dwell in awe. This prompted people to watch an awe-inspiring video on Yosemite and reflect on what they liked and felt when watching it. (Takes approximately 6 min)
- Be a force of good. This prompted people to listen to an audio-guided reflection on how they can contribute goodness to the world. (Takes approximately 8 min)

These brief well-being activities were drawn from the Greater Good in Action digital repository of research-based practices and modified for brevity and suitability for a digital platform. They are all publicly available to anyone with internet access.

### Outcome Measures

#### Overview

Before and after the 7-day program, participants completed an identical battery of measures related to their well-being and health. Participants were asked to think back on the past week for all outcomes. The battery had 18 items selected to minimize participant burden and match the “brief” tone of the Big Joy Project. These outcome measures were drawn from existing well-being research and have been used in prior large-scale studies. Measures were selected for their brevity and face validity; they demonstrated usefulness in digital and population-based research. No new scales or items were developed specifically for this study. Prior research has shown that single items have been documented to have similar predictive power as multiitem measures [44,45]. In this analysis, we focused on 6 outcomes. The first 3 were emotional well-being, positive emotions, and happiness agency, which the Big Joy Projected intended to increase. We also reported findings on 3 health-related outcomes: stress, self-reported health, and sleep quality.

#### Emotional Well-Being (3 Items)

Emotional well-being consists of a person’s overall evaluation of life, typical emotional experiences, and sense of meaning or purpose in life [7]. Emotional well-being was measured with 3 items in which participants rated how much they agreed with the following statements on a 0 (strongly disagree) to 10 (strongly agree) Likert scale: “I feel satisfied with my life as a whole,” “I usually feel happy,” and “I feel that the things that I do in my life are worthwhile.” The 3 items were averaged to measure emotional well-being (baseline: n=17,598; α=.88; mean 6.09, SD 2.29).

#### Positive Emotions (3 Items)

Positive emotions were measured with 3 items. Participants rated how much they agreed with the following statements on a 0 (strongly disagree) to 10 (strongly agree) Likert scale [46]: “I feel hopeful, optimistic, or encouraged,” “I feel wonder, amazement, or awe,” and “I feel amused, fun loving, or silly. We originally intended to capture the experience of discrete positive emotions [47] and analyze them separately. However, because the results were similar and items highly correlated, we decided to average them to measure experiences of discrete positive emotions (baseline: n=17,625; α=.86; mean 5.98, SD 2.26).

#### Happiness Agency (1 Item)

Happiness agency refers to a person’s self-perception of how much they can influence their happiness. Happiness agency was measured with 1 item in which participants rated how much they agreed with the following statement on a 0 (strongly disagree) to 10 (strongly agree) Likert scale: “I feel able to impact, influence, or play an active role in how happy I generally feel” (baseline: n=17,692; mean 5.82, SD 2.71).

#### Stress, Self-Reported Health, and Sleep Quality (1 Item Each)

Stress was measured with 1 item in which participants rated how much they agreed with the following statement on a 0 (strongly disagree) to 10 (strongly agree) Likert scale: “I have felt stressed, nervous, or overwhelmed” (baseline: n=17,700; mean 5.82, SD 2.71). Self-reported health and sleep were measured with participants’ replies to “How would you rate your physical health?” (baseline: n=17,756; mean 3.24, SD 0.98) and “How would you rate the average quality of your sleep?” (baseline: n=17,757; mean 2.76, SD 1.02) on a 1 (poor) to 5 (excellent) Likert scale.

### Moderators

As part of the primary analysis, we also tested whether number of activities completed (dosage) moderated the intervention effects. For each daily activity prompt, participants rated their feelings before and after. An activity was operationalized as completed if a participant answered both the preprompt and postprompt questions. Some participants may have completed some or all the intervention activities without submitting responses; however, because the activities were self-administered on the web without direct supervision, we could not objectively verify engagement. Therefore, we used submission of both preprompt and postprompt responses as an imperfect but conservative and standardized behavioral proxy for activity completion. Dosage was calculated as the number of activities completed, ranging from 0 to 7.

We then tested 6 sociodemographic and psychosocial variables. We targeted the following key sociodemographic variables: age, sex, race and ethnicity, and educational level; in addition, we targeted moderators that capture participants’ socioeconomic status: subjective social status and financial strain.

Age was calculated using the month and year, and sex was measured by selecting from the following options: female, male, nonbinary, or other, with an open-ended text option. Due to the small number of participants who selected nonbinary and other, these 2 categories were combined into nonbinary.

For race and ethnicity, participants selected a specific group from 7 broad categories: Black, African, and Caribbean; Latin American and Hispanic; White and European; Asian; Middle Eastern and Arab; Indigenous; and prefer not to say. For example, Black, African, and Caribbean contained the following subgroups: Black, North African, Central African, South African, East African, West African, and Caribbean. Although each subgroup is unique, we collapsed them into their broader category to obtain sufficient subgroup sample sizes. Participants also had the option to select more than 1 identity, so we added a mixed category.

For education level, people selected 1 option from 10 categories: less than grade 9, more than grade 9 but less than grade 12, high school graduate or equivalent, trade or vocational training, associate degree, bachelor’s degree, graduate degree, professional degree, doctoral degree, and other. We collapsed responses into 3 categories: high school or less, college graduate or some college, and graduate degree. The category “other” likely indicated some type of higher educational degree or certificate, so we included it in the middle category, such as college graduate or some college.

Subjective social status was measured with the MacArthur Subjective Social Status ladder, which asked participants to imagine that the ladder represents how their society is set up. To make this item more straightforward and applicable across cultures, we altered and expanded the wording of the question. We stated, “At the top of the ladder are the people with the most privilege, the money, education, and access to the most sought-after jobs. At the bottom are the people with the least privilege, money, education, and access to sought-after employment or no job” [48]. Participants indicated where they ranked on the ladder on a scale of 0 to 10.

Finally, participants rated their financial strain with the question, “In the past month, it has been difficult for me to pay for the very basics like food, housing, medical care, and heating or air conditioning?” on a scale of 1 (strongly disagree) to 5 (strongly agree). Due to a coding error on our website, the anchors were 0 to 10 before June 8, 2023, before switching them to a 1 to 5 scale. Therefore, for the financial strain moderator analysis, we excluded cases after June 8, 2023, resulting in a smaller sample size of 9,386 for this test.

### Statistical Analysis

All models were conducted using the *lme4* package in R (R Foundation for Statistical Computing). For each outcome, we specified a linear mixed-effects model with a fixed effect of time (pre-post), relevant moderators (eg, age and financial strain), and interaction terms (eg, time × moderator). Continuous moderator variables were centered to facilitate the interpretation of pre-post main effects. Participant ID was included as a random intercept to account for the nested structure of repeated measures.

To evaluate the pre-post impact of the 7-day Big Joy intervention, we conducted several mixed-effects models with dosage as a moderator. We treated dosage as a continuous variable and probed each simple slope using the emmeans package at +1 or –1 SD for significant interactions (Multimedia Appendix 1)*.* There were 385 to 407 people (sample size varied depending on the outcome) who did not engage in any daily activities but completed the pre- and postintervention surveys. Because this group acts as a natural comparison that provides a quasi-control condition, we also included an analysis of dose as a factorial variable in Multimedia Appendix 2.

We then tested whether the pre-post intervention effects were moderated by the following sociodemographic and psychosocial factors: age, sex, race and ethnicity, educational level, subjective social status, and financial strain. For ease of presentation, we present results of continuous moderators, such as age, subjective social status, and financial strain, and categorical moderators, such as sex, race and ethnicity, and educational level in separate tables. We conducted separate mixed-effects models to predict if the pre-post intervention effects interacted with each moderator. For significant interactions of models that used a continuous moderator, we report *t* statistics. We probed each simple slope using the emmeans package at +1 or –1 SD for significant interactions with continuous moderators. For subjective social status, we also controlled for education level, consistent with the literature [49].

For categorical moderators, we used and reported an *F* test for significant interactions and used the Wilcoxon rank sum test (*W*) to compare the difference scores with a reference group due to non-normal distributions and unequal group sizes. For sex, the reference group was female; for race and ethnicity, the reference group was White; for educational level, the reference group was high school or less. We applied the Benjamini-Hochberg procedure for all post hoc analyses to account for multiple comparisons. The Benjamini-Hochberg procedure helps control false discovery rates by sorting *P* values from smallest to largest and adjusting them based on their ranking [50]. Smaller *P* values are held to stricter standards, while larger *P* values are given more thresholds. We report adjusted *P* values in the tables. Finally, we also examined the correlation between the intercept and pre-post intervention slopes for each model to assess whether people with lower baseline scores (higher for the stress outcome) benefited more from the intervention. Multimedia Appendix 3 provides more details.

## Results

### Participants

A total of 48,789 participants consented to the study, and 17,598 (36%) participants completed both pre-post weekly emotional well-being measures. Only those who completed both pre-post measures were included in the analysis. The sample size and missing values varied depending on the variables used for each analysis. The demographics in Table 1 included a sample size for participants who reported pre-post weekly emotional well-being measures, age, sex, ethnicity, and education (n=17,434). Participants were older, with a mean age of 54.0 (SD 14.0) years, predominantly female (14,632/17,434, 83.93%), White (12,886/17,434, 73.91%), and completed a graduate degree (9034/17,434, 51.82%). In total, 169 countries were represented in our sample. A majority of the sample were from the United States (9122/17,434, 52.32%), Canada (1846/17,434, 10.59%), and Great Britain (1314/17,434, 7.54%). In total, 26 countries had ≥50 participants.

**Table 1:** Demographic characteristics of participants.

Demographics		Values
**Age (y)**		
	Mean (SD)	54.03 (14.02)
	Median (IQR; range)	55 (45-64; 18-103)
**Sex, n (%)**		
	Female	14,632 (83.93)
	Male	2612 (14.98)
	Nonbinary	190 (1.09)
**Ethnicity, n (%)**		
	Asian	1319 (7.57)
	Black	831 (4.77)
	Latinx	711 (4.08)
	Middle Eastern	73 (0.42)
	White	12,886 (73.91)
	Mixed	1111 (6.37)
	Native	53 (0.3)
	Prefer not to say	450 (2.58)
**Education, n (%)**		
	High school or less	1353 (7.76)
	College graduate or some college	7047 (40.42)
	Graduate degree	9034 (51.82)

### Intervention Results

Table 2 presents pre-post outcomes. The sample size varied slightly depending on the outcome, ranging from 17,598 to 17,757. Participants reported an increase in emotional well-being (t_17,600_=78.68; β=1.05; *P<*.001; *d_z_*=0.48), positive emotions (t_17,620_=69.41; β=0.95; *P<*.001; *d_z_*=0.45), and happiness agency (t_17,690_=61.25; β=0.97; *P<*.001; *d_z_*=0.44) after the intervention. Moreover, participants reported a decrease in perceived stress (t_17,700_=–46.73, β=–0.96; *P<*.001; *d_z_*=–0.35) and an increase in self-reported health (t_17,750_=15.20; β=0.07; *P<*.001; *d_z_*=0.07) and sleep quality (t_17,760_=27.84; β=0.15; *P<*.001; *d_z_*=0.15) after the intervention.

**Table 2:** Intervention effects on pre-post outcomes related to well-being and health.

Outcomes	Pre, mean (SD)	Post, mean (SD)	Sample size, n	*d_z_^a^*	*P* value
Emotional well-being	6.09 (2.29)	7.14 (1.97)	17,598	0.48	<.001
Positive emotions	5.98 (2.26)	6.93 (1.95)	17,625	0.45	<.001
Happiness agency	6.43 (2.38)	7.40 (2.04)	17,692	0.44	<.001
Perceived stress	5.82 (2.71)	4.85 (2.70)	17,700	–0.35	<.001
Self-reported health	3.24 (0.98)	3.31 (0.96)	17,756	0.07	<.001
Sleep quality	2.76 (1.02)	2.90 (0.99)	17,757	0.15	<.001

^a^*d_z_* represents within-person Cohen *d*.

The intervention effects were moderated by dosage or number of completed interventions on all outcomes (*P* values<.004). Participants completed a mean of 5.17 (SD 1.85) interventions for emotional well-being. We report interaction *t* statistics and corresponding interaction β values. Simple slopes analysis shows that people who completed more of the intervention days (+1 SD), compared to those who completed fewer (–1 SD), reported larger increases in emotional well-being (β=1.23 vs β=0.87; t_17,600_=13.49; interaction β=0.10; *P<*.001), positive emotions (β=1.15 vs β=0.75; t_17,620_=14.52; interaction β=0.11; *P<*.001), happiness agency (β=1.21 vs β=0.74; t_17,690_=14.85; interaction β=0.13; *P*<.001), self-reported health (β=0.09 vs β=0.05; t_17,750_=4.77; interaction β=0.01; *P*<.001), and sleep quality (β=0.16 vs β=0.13; t_17,760_=2.95; interaction β=0.01; *P*=.003). People who completed more intervention days (+1 SD), compared to those who completed fewer (–1 SD), also reported larger reductions in stress (β=–1.10 vs β=–0.82; t_17,700_=–6.90; interaction β=0.08; *P*<.001). Multimedia Appendix 1 provides more details. Interestingly, even people who completed the pre- and postweekly assessment but reported not engaging in any brief interventions (dose 0) experienced a small increase in most outcomes (*d_z_*=|0.11 to 0.23|), except self-reported health, as shown in Multimedia Appendix 2.

### Moderator Analyses

Next, we tested how key sociodemographic factors moderated the intervention effects.

#### Age

Age significantly moderated the effects of the Big Joy intervention on emotional well-being (*P*<.001), positive emotions (*P*=.02), happiness agency (*P*=.004), perceived stress (*P*<.001), and sleep quality (*P*<.001), but not self-reported health (*P*=.07). Simple slope analysis shows that younger people (–1 SD), compared to older people (+1 SD), reported larger increases in emotional well-being (β=1.16 vs β=0.94; t_17,600_=–8.30; β=–0.01; *P*<.001), positive emotions (β=0.98 vs β=0.92; t_17,620_=–2.28; β=–0.002; *P*=.02), happiness agency (β=1.02 vs β=0.92; t_17,690_=–2.89; β=–0.003; *P*=.004), and sleep quality (β=0.17 vs β=0.13; t_17,760_=–3.59; β=–0.001; *P*<.001). Younger people also reported a larger decrease in perceived stress (β=–1.04 vs β=–0.89; t_17,700_=3.66; β=–0.005; *P*<.001) compared to older people (+1 SD; Table 3).

**Table 3:** Continuous moderators: age, financial strain, and subjective social status.

Outcomes		Age		Financial strain		Subjective social status	
		β (SE)	*P* value	β (SE)	*P* value	β (SE)	*P* value
**Emotional well-being**							
	Mean (–1 SD)	1.16 (0.02)	<.001	0.83 (0.03)	<.001	1.36 (0.02)	<.001
	Mean (+1 SD)	0.94 (0.02)	<.001	1.40 (0.03)	<.001	0.73 (0.02)	<.001
**Positive emotions**							
	Mean (–1 SD)	0.98 (0.02)	<.001	0.81 (0.03)	<.001	1.19 (0.02)	<.001
	Mean (+1 SD)	0.92 (0.02)	<.001	1.13 (0.03)	<.001	0.71 (0.02)	<.001
**Happiness agency**							
	Mean (–1 SD)	1.02 (0.02)	<.001	0.82 (0.03)	<.001	1.21 (0.02)	<.001
	Mean (+1 SD)	0.92 (0.02)	<.001	1.15 (0.03)	<.001	0.73 (0.02)	<.001
**Perceived stress**							
	Mean (–1 SD)	–1.04 (0.03)	<.001	–0.86 (0.04)	<.001	—^a^	—
	Mean (+1 SD)	–0.89 (0.03)	<.001	–1.10 (0.04)	<.001	—	—
**Self-reported health**							
	Mean (–1 SD)	0.08 (0.01)	<.001	0.03 (0.01)	.003	0.10 (0.01)	<.001
	Mean (+1 SD)	0.06 (0.01)	<.001	0.13 (0.01)	<.001	0.03 (0.01)	<.001
**Sleep quality**							
	Mean (–1 SD)	0.17 (0.01)	<.001	0.10 (0.01)	<.001	0.19 (0.01)	<.001
	Mean (+1 SD)	0.13 (0.01)	<.001	0.21 (0.01)	<.001	0.10 (0.01)	<.001

^a^Nonsignificant interaction with the moderator.

#### Sex

Sex did not significantly moderate the effects of the Big Joy program on any outcomes (*P*>.05).

#### Race and Ethnicity

Race and ethnicity significantly moderated the effects of the Big Joy program on emotional well-being (*F*_7,17,534_=29.05; *P*<.001), positive emotions (*F*_7,17,562_=9.45; *P*<.001), happiness agency (*F*_7,17,627_=5.17; *P*<.001), self-reported health (*F*_7,17,693_=9.02; *P*<.001), and sleep quality (*F*_7,17,694_=6.05; *P*<.001), but not perceived stress (*F*_7,17,635_=1.29; *P*=.25). Using those who identified as White as a reference group, those who identified as Black reported larger increases in emotional well-being (*d_z_*=0.46 vs *d_z_*=0.77*; W*=6,536,890; *P*<.001), positive emotions (*d_z_*=0.44 vs *d_z_*=0.59; *W*=6,058,593; *P*<.001), happiness agency (*d_z_*=0.43 vs *d_z_*=0.53; *W*=5,976,286; *P*=.002), self-reported health (*d_z_*=0.06 vs *d_z_* =0.18; *W*=6,229,975; *P*<.001), and sleep quality (*d_z_*=0.13 vs *d_z_*=0.26; *W*=6,263,986; *P*<.001). The sample sizes varied by outcomes (range 12,973-13,014 for White participants and 838-883 for Black participants).

Those who identified as Asian (range 1322-1342) reported larger increases in health (*d_z_*=0.06 vs *d_z_*=0.10; *W*=9,002,168; *P=*.03). Those who identified as Latinx (range 717-742) also reported larger increases in emotional well-being (*d_z_*=0.46 vs *d_z_*=0.57; *W*=5,041,159; *P*=.001) and happiness agency (*d_z_*=0.43 vs *d_z_*=0.49; *W*=4,986,750; *P*=.02). Those who identified as mixed (range 1113-1146) also reported larger increases in emotional well-being (*d_z_*=0.46 vs *d_z_*=0.53; *W*=7,578,367; *P*=.01) and self-reported health (*d_z_*=0.06 vs *d_z_*=0.13; *W*=7,792,832; *P*=.004). Tables 4 and 5 provide details about participants who preferred not to disclose their race and ethnicity. There were no other significant comparisons. Although the effect size estimates for Native and Indigenous and Middle Eastern participants were large, they did not differ significantly from those of White participants. This may be due to the lower statistical power. We had <100 people in Native and Indigenous and Middle Eastern participants (Tables 4 and 5).

**Table 4:** Moderator analysis by race and ethnicity.

Race and ethnicity	Sample size, n^a^ (%)	Emotional well-being			Positive emotions			Happiness agency	
		*d_z_^b^*	*P* value vs reference^c^	*d_z_*		*P* value vs reference	*d_z_*		*P* value vs reference
White (reference)	12,973	0.46^d^	—^e^	0.44^d^		—	0.43^d^		—
Black	838	0.77^d^	<.001	0.59^d^		<.001	0.53^d^		.002
Asian	1322	0.47^d^	.49	0.40^d^		.19	0.41^d^		.97
Latinx	717	0.57^d^	.001	0.53^d^		.10	0.49^d^		.02
Native	53	0.52^d^	.98	0.25^f^		.16	0.38^g^		.97
Middle Eastern	73	0.56^d^	.74	0.58^d^		.33	0.41^d^		.97
Mixed	1113	0.53^d^	.01	0.48^d^		.12	0.42^d^		.97
Prefer not to say	453	0.45^d^	.60	0.40^d^		.51	0.42^d^		.97

^a^n based on emotional well-being sample.

^b^*d_z_* represents within-person Cohen *d*.

^c^*P* value versus reference represents adjusted *P* values compared to the reference group using the Wilcoxon rank sum test and Benjamini-Hochberg corrections.

^d^*P*<.001 for pre-post values.

^e^No values because it is the reference group.

^f^Nonsignificant pre-post effects.

^g^*P*<.05 for pre-post values.

**Table 5:** Moderator analysis by race and ethnicity.

Race and ethnicity	Sample size, n^a^ (%)	Perceived stress			Self-reported health			Sleep quality	
		*d_z_^b^*	*P* value vs reference^c^	*d_z_*		*P* value vs reference	*d_z_*		*P* value vs reference
White (reference)	13,019	–0.36^d^	N/A^e^	0.06^d^		N/A	0.13^d^		N/A
Black	856	–0.31^d^	—^g^	0.18^d^		<.001	0.26^d^		<.001
Asian	1334	–0.34^d^	—	0.10^d^		.03	0.16^d^		.32
Latinx	724	–0.43^d^	—	0.08^g^		.38	0.16^d^		.32
Native	54	–0.40^g^	—	0.08^h^		.90	0.04^h^		.32
Middle Eastern	74	–0.30^g^	—	<0.01^h^		.50	0.04^h^		.32
Mixed	1123	–0.35^d^	—	0.13^d^		.004	0.18^d^		.32
Prefer not to say	459	–0.31^d^	—	0.15^d^		.005	0.15^d^		.64

^a^n based on perceived stress sample.

^b^*d_z_* represents within-person Cohen *d*.

^c^*P* value versus reference represents adjusted *P* values compared to the reference group using the Wilcoxon rank sum test and Benjamini-Hochberg corrections.

^d^*P*<.001 for pre-post values.

^e^N/A: not applicable (no values because it is the reference group).

^f^Nonsignificant interaction with the moderator.

^g^*P*<.01 for pre-post values.

^h^Nonsignificant pre-post effects.

#### Educational Level

Educational level significantly moderated the effects of the Big Joy program on emotional well-being (*F*_2,17,570_=47.51; *P*<.001), positive emotions (*F*_2,17,595_=20.32; *P*<.001), happiness agency (*F*_2,17,664_=9.52; *P*<.001), self-reported health (*F*_2,17,735_=14.67; *P*<.001), and sleep quality (*F*_2,17,570_=47.51; *P*<.001), but not perceived stress (*F*_2,17,672_=2.38; *P*=.09). Using people who reported at least a high school education (range 1370-1417) as a reference group, people with some college (range 7101-7167) had lower increases in emotional in emotional well-being (*d_z_*=0.56 vs *d_z_*=0.52; *W*=4,585,954; *P*=.001), self-reported health (*d_z_*=0.13 vs *d_z_*=0.09; *W*=4,936,091; *P*=.04), and sleep quality (*d_z_*=0.21 vs *d_z_*=0.15; *W*=4,862,084; *P*=.004). Moreover, using people who reported at least a high school education as a reference group, people with a graduate degree had lower increases in emotional well-being (*d_z_*=0.56 vs *d_z_*=0.45; *W*=5,484,452; *P*<.001), positive emotions (*d_z_*=0.49 vs *d_z_*=0.42; *W*=5,484,452; *P<*.001), self-reported health (*d_z_*=0.13 vs *d_z_*=0.05; *W*=6,154,751; *P*<.001), and sleep quality (*d_z_*=0.21 vs *d_z_*=0.13; *W*=6,148,074; *P*=.001). Although education significantly moderated the intervention effects on happiness agency, the comparison with high school as a reference was not significant (Tables 6 and 7).

**Table 6:** Moderator analysis by education.

Education	Sample size, n^a^ (%)	Emotional well-being			Positive emotions			Happiness agency	
		*d_z_^b^*	*P* value vs reference^c^	*d_z_*		*P* value vs reference	*d_z_*		*P* value vs reference
High school or less (reference)	1370	0.56^d^	—^e^	0.49^d^		—	0.42^d^		—
Some college or college graduate	7101	0.52^d^	.001	0.47^d^		.14	0.46^d^		.85
Graduate degree	9102	0.45^d^	<.001	0.42^d^		<.001	0.42^d^		.06

^a^n based on emotional well-being and perceived stress samples, respectively.

^b^*d_z_* represents within-person Cohen *d*.

^c^*P* versus reference represents adjusted *P* values compared to the reference group using the Wilcoxon rank sum test and Benjamini-Hochberg corrections.

^d^*P*<.001 for pre-post values.

^e^No values because it is the reference group.

**Table 7:** Moderator analysis by education.

Education	Sample size, n^a^ (%)	Perceived stress			Self-reported health			Sleep quality	
		*d_z_^b^*	*P* value vs reference^c^	*d_z_*		*P* value vs reference	*d_z_*		*P* value vs reference
High school or less (reference)	1390	–0.27^d^	N/A^e^	0.13^d^		N/A	0.21^d^		N/A
Some college or college graduate	7140	–0.36^d^	—^f^	0.09^d^		.04	0.15^d^		.004
Graduate degree	9145	–0.36^d^	—	0.05^d^		<.001	0.13^d^		.001

^a^n based on emotional well-being and perceived stress samples, respectively.

^b^*d_z_* represents within-person Cohen *d*.

^c^*P* versus reference represents adjusted *P* values compared to the reference group using the Wilcoxon rank sum test and Benjamini-Hochberg corrections.

^d^*P*<.001 for pre-post values.

^e^N/A: no values because it is the reference group.

^f^Nonsignificant interaction with the moderator.

#### Financial Strain

Financial strain significantly moderated the effects of the Big Joy program on emotional well-being (t_9384_=14.90; interaction β=0.23; *P*<.001), positive emotions (t_9394_=8.20; interaction β=0.13; *P*<.001), happiness agency (t_9436_=7.25; interaction β=0.13; *P*<.001), perceived stress (t_9436_=–3.98; interaction β=–0.09; *P*<.001), self-reported health (t_9445_=7.57; interaction β=0.04; *P*<.001), and sleep quality (t_9432_=7.32; interaction β=0.04; *P*<.001). Simple slope analyses showed that people who reported higher financial strain (+1 SD), compared to those with lower financial strain (–1 SD), had a larger increase in emotional well-being (β=1.40 vs β=0.83; *P*<.001), positive emotions (β=1.13 vs β=0.81; *P*<.001), happiness agency (β=1.15 vs β=0.82; *P*<.001), self-reported health (β=0.13 vs β=0.03; *P*<.001; *P*<.001), and sleep quality (β=0.21 vs β=0.10; *P*<.001). Participants with higher financial strain (+1 SD) also reported larger decreases in stress (β=–1.10 vs β=–0.86; *P*<.001) compared to those with lower financial strain (Table 3).

#### Subjective Social Status

Subjective social status significantly moderated the effects of the Big Joy program on emotional well-being (t_17,410_=–24.03; interaction β=–0.17; *P*<.001), positive emotions (t_17,410_=–17.38; interaction β=–0.13; *P*<.001), happiness agency (t_17,470_=–15.14; interaction β=–0.13; *P*<.001), self-reported health (t_17,440_=–7.38; interaction β=–0.02; *P*<.001), and sleep quality (t_17,440_=–8.24; interaction β=–0.02; *P*<.001), but not perceived stress (*P*=.13). Simple slope analyses show that people who report lower subjective social status (–1 SD), compared to those who reported higher subjective social status, had a larger increase in emotional well-being (β=1.36 vs β=0.73; *P*<.001), positive emotions (β=1.19 vs β=0.71; *P*<.001), happiness agency (β=1.21 vs β=0.73; *P*<.001), self-reported health (β=0.10 vs β=0.03; *P*<.001), and sleep quality (β=0.19 vs β=0.10; *P*<.001; Table 3).

## Discussion

### Principal Findings

We found evidence that participating in the Big Joy program, a brief web-based intervention for boosting emotional well-being, significantly increased well-being outcomes while decreasing perceived stress. Specifically, we found increases in emotional well-being (*d_z_*=0.48), positive emotions (*d_z_*=0.45), and perceived agency over happiness (*d_z_*=0.44). Remarkably, the effect sizes for these outcomes are comparable to more extensive multicomponent studies. For example, the estimated pre-post effect size for well-being is approximately *d_z_*=0.35 for the 5waysA [34], *d_z_*=0.67 for the ENHANCE program [32], and *d_z_*=0.37 for the abridged ENHANCE program [33]. It is important to note that these more complex, multicomponent studies are conducted over longer periods. For example, the 5waysA program takes 10 weeks [34], and the ENHANCE program takes 12 weeks [32].

Participation in the Big Joy program also reduced perceived stress and improved self-reported physical health and sleep quality, important markers for long-term health. Increased stress has long been implicated with poorer health, such as impaired immune system [51,52], shorter telomere length [53-55], and an increased risk for cardiovascular [56] and other diseases [57]. Although our health-related questions are self-reported, these measures are highly associated with objective health measures [58,59]. For example, higher self-reported health is associated with lower mortality risk [60-62]. Self-reported sleep quality is positively related to quality of life [63] and reduced risk for coronary heart disease and all-cause mortality [64]. Although our effect sizes for changes in self-reported health and sleep quality are small, they can be meaningful at the population level [65,66]. For example, associations between aspirin and the prevention of heart attacks (*r*=0.03) or between patient education and exercise among people with cardiac conditions (*r*=0.09) are small [67] according to the guidelines by Cohen [68]; however, they are meaningful from a public health perspective [65].

Not surprisingly, those who engaged in more interventions tended to benefit more. Moreover, participating in as few as 3 interventions was enough to significantly increase emotional well-being compared to those who did not engage in any.

### Sociodemographic Moderators

Our large-scale study also allowed us to analyze critical sociodemographic variables that could potentially impact the effectiveness of our intervention. First, younger people, compared to older ones, had a larger increase in emotional well-being, positive emotions, happiness agency, and sleep quality. Younger people also had a larger decrease in stress compared to older people. This finding contrasts with a recent meta-analysis that reported greater improvements in well-being among older adults compared to those in earlier stages of life [25]. Older adults tend to have higher baseline emotional well-being in our sample than younger people, which may help explain the greater benefit for young adults in our study.

Sex did not moderate any intervention effects. This is consistent with a meta-analysis showing that the percentage of female participants does not moderate positive psychology intervention effects [25].

Contrary to the meta-analysis by Carr et al [25], race and ethnicity moderated the pre-post intervention effects on emotional well-being, positive emotions, happiness agency, self-reported health, and sleep quality, but not perceived stress. Using those who reported as White as the reference group, those who identified as Black reported larger increases in emotional well-being and all other outcomes compared to their White counterparts. Those who identified as Latinx also reported larger increases in emotional well-being and happiness agency than those who identified as White.

We also tested a set of moderators that reflected socioeconomic status: educational level, subjective social status, and financial strain. They all paint a similar pattern: people who are less privileged benefit more from the intervention. For example, people who reported only having a high school education had larger increases in emotional well-being, positive emotions, happiness agency, self-reported health, and sleep quality than those with graduate degrees. Those with high school education also had larger increases in emotional well-being, self-reported health, and sleep quality than those with college-level education.

A similar pattern was found in subjective social status. Those who reported lower subjective social status had a larger increase in emotional well-being, positive emotions, happiness agency, self-reported health, and sleep quality than those who reported higher subjective social status. Finally, people with higher financial strain report more significant increases in emotional well-being, positive emotions, happiness agency, self-reported health, and sleep quality.

We identified a pattern of greater benefits in our sociodemographic groups that are less privileged. To better understand why participants from less privileged backgrounds showed greater improvements, we examined the correlation between baseline scores (intercepts) and the pre-post intervention slopes. We found negative correlations across outcomes, suggesting that people who began with lower well-being (or higher stress) experienced greater benefits. This pattern helps, in part, to explain the stronger intervention effects among people who are less privileged. Multimedia Appendix 3 provides more details. Nevertheless, these patterns are particularly noteworthy because people with less privilege are more at risk for poor health outcomes. For example, people with lower levels of educational attainment tend to have elevated levels of interleukin-6, an inflammatory marker implicated in numerous diseases [69]. Higher well-being tends to buffer these adverse effects [69]. Interestingly, we found that people with less privilege are more likely to drop out of this intervention [37], even if they may benefit the most.

### Strengths and Limitations

Our study has several strengths. First, we have a large and global sample. Although our population sample was predominantly White and highly educated, we still had enough variability to test key sociodemographic moderators. Second, our intervention was designed to be brief and not burdensome compared to other multicomponent interventions that span 10 to 12 weeks and take approximately 3 to 4 hours a week to complete [32,34]. The Big Joy Project takes approximately 5 to 10 minutes daily for 7 days. Third, our study was web-based, free of cost, and highly accessible to anyone with internet access. This makes the Big Joy Project highly scalable and can be done by anyone with internet access.

Our study also has several limitations. First, we do not have a control group for comparison, so we cannot make between-subject comparisons with people in a structurally equivalent control group. Although we had participants who signed up for the study but did not engage in any activities (operationalized as completing the daily brief pre- and postintervention questions) as a quasi-control group (n=385 to 407 depending on the outcome), we recognize that this is not equivalent to a randomized control group. Therefore, we cannot make causal conclusions. However, given the truncated timeline of 1 week, we did not expect emotional well-being and other outcome measures to change significantly in a short time. Evidence suggests that emotional well-being is relatively stable in short periods [70,71]. The nonengaged participants improved in nearly every outcome, although to a smaller degree than those who engaged in the daily interventions (Multimedia Appendix 2). This suggests that the well-being practices led to improved well-being and health-related outcomes. Nevertheless, future research should compare brief well-being intervention programs to a structurally equivalent control group.

Another limitation is self-selection bias because it was an opt-in community sample. The Big Joy Project was advertised through various social media outlets and partner organizations, and participants required access to a web-based platform*.* Therefore, participants were likely more tech-savvy, psychologically motivated, and already interested in well-being interventions. This could have overestimated the effectiveness of the intervention and limited the generalizability of our results. Moreover, participants may have increased expectations of positive outcomes. Indeed, people who signed up for the study but did not engage in any activities showed a slight increase in emotional well-being (*d_z_*=0.19). Nevertheless, we tried to minimize positive expectation effects by framing the study as a way for people to figure out what well-being intervention works for them as opposed to a well-being intervention that is going to boost their well-being. Future research should test this brief intervention in a large-scale randomized controlled study using more diverse recruitment strategies to better assess generalizability.

One important limitation is that we did not include a follow-up assessment to examine whether improvements in well-being persisted over time. In comparison, people in the ENHANCE program [32] maintained increased well-being 3 months after the intervention. Without follow-up data, it remains unclear whether the well-being benefits observed in Big Joy are sustained over time or represent short-term effects. Future research should assess the durability of Big Joy’s effects and examine whether habit-formation strategies are needed to maintain benefits.

Another limitation concerns the financial strain moderator analyses. Due to a midstudy change in the financial strain measure (from 0-10 to 1-5), participants who enrolled after June 8, 2023, were excluded from these analyses. This exclusion was necessary to maintain measurement validity, although it did reduce the sample size for the financial strain moderation test and may have introduced bias if the excluded participants differed systematically from those included in the analyses. Future studies should ensure consistency in measurement across the study to avoid this limitation and maximize statistical power.

Finally, to reduce participant burden and recruit more participants, we used measures that required fewer items, with some constructs only being measured by 1 item (happiness agency, stress, self-reported health, and sleep quality). Our emotional well-being and positive emotions measures were comparable to existing measures of ≥3 items and had high reliability. Nevertheless, prior research has shown that single items perform as adequately as their corresponding lengthier measures [44,72]. Future studies may want to use more extensive measures for brief well-being intervention studies.

### Conclusions

Our study provides evidence that a brief web-based well-being intervention, the Big Joy Project, is an effective and promising intervention for increasing well-being and health-related outcomes. Moreover, our study identifies key sociodemographic variables that moderate these intervention effects. For example, younger people benefit more from the Big Joy Project than older people. People who identify as Black seem to benefit more than those who identify as White. Socioeconomic status variables also moderate our intervention effects. Those with lower education, those who report lower subjective social status, and those with more financial strain benefit more from the interventions than those with higher education, those who report higher subjective social status, and those with less financial strain. This study offers a practical, scalable, low-cost web-based intervention to promote well-being and health. Moreover, the Big Joy Project appears to be more effective for people who may benefit from it the most.

## Data Availability

The data used in this study are not publicly available because participants did not provide explicit consent for data sharing. Access to the data may be considered upon reasonable request and pending ethical approval.

## Appendix

Multimedia Appendix 1 Table displaying moderation by dose as continuous variables.

Multimedia Appendix 2 Table displaying moderation by dose as categorical variables.

Multimedia Appendix 3 Table displaying intercept and pre-post slope correlations.

## Abbreviations

5waysA: Five Ways to Wellbeing for All
ENHANCE: Enduring Happiness and Continued Self-Enhancement

## References

[1] (2011). National prevention strategy: America’s plan for better health and wellness. US Department of Health and Human Services, Office of the Surgeon General.

[2] Keyes CL (2002). The mental health continuum: from languishing to flourishing in life. J Health Soc Behav.

[3] Keyes CL, Dhingra SS, Simoes EJ (2010). Change in level of positive mental health as a predictor of future risk of mental illness. Am J Public Health.

[4] Chida Y, Steptoe A (2008). Positive psychological well-being and mortality: a quantitative review of prospective observational studies. Psychosom Med.

[5] Howell RT, Kern ML, Lyubomirsky S (2007). Health benefits: meta-analytically determining the impact of well-being on objective health outcomes. Health Psychol Rev.

[6] Feller SC, Castillo EG, Greenberg JM, Abascal P, Van Horn R, Wells KB, University of California‚ Los Angeles Community Translational Science Team (2018). Emotional well-being and public health: proposal for a model national initiative. Public Health Rep.

[7] Park CL, Kubzansky LD, Chafouleas SM, Davidson RJ, Keltner D, Parsafar P, Conwell Y, Martin MY, Hanmer J, Wang KH (2023). Emotional well-being: what it is and why it matters. Affect Sci.

[8] Kubzansky LD, Kim ES, Boehm JK, Davidson RJ, Huffman JC, Loucks EB, Lyubomirsky S, Picard RW, Schueller SM, Trudel-Fitzgerald C, VanderWeele TJ, Warran K, Yeager DS, Yeh CS, Moskowitz JT (2023). Interventions to modify psychological well-being: progress, promises, and an agenda for future research. Affect Sci.

[9] Jiwani Z, Tatar R, Dahl CJ, Wilson-Mendenhall CD, Hirshberg MJ, Davidson RJ, Goldberg SB (2023). Examining equity in access and utilization of a freely available meditation app. Npj Ment Health Res.

[10] Bryan CJ, Tipton E, Yeager DS (2021). Behavioural science is unlikely to change the world without a heterogeneity revolution. Nat Hum Behav.

[11] Pressman SD, Jenkins BN, Moskowitz JT (2019). Positive affect and health: what do we know and where next should we go?. Annu Rev Psychol.

[12] Steptoe A, Deaton A, Stone AA (2015). Subjective wellbeing, health, and ageing. Lancet.

[13] Grant CA, Wallace LM, Spurgeon PC (2013). An exploration of the psychological factors affecting remote e‐worker's job effectiveness, well‐being and work‐life balance. Empl Relat.

[14] Wood AM, Joseph S (2010). The absence of positive psychological (eudemonic) well-being as a risk factor for depression: a ten year cohort study. J Affect Disord.

[15] Schotanus-Dijkstra M, Drossaert CH, Pieterse ME, Boon B, Walburg JA, Bohlmeijer ET (2017). An early intervention to promote well-being and flourishing and reduce anxiety and depression: a randomized controlled trial. Internet Interv.

[16] Schotanus-Dijkstra M, Ten Have M, Lamers SM, de Graaf R, Bohlmeijer ET (2017). The longitudinal relationship between flourishing mental health and incident mood, anxiety and substance use disorders. Eur J Public Health.

[17] Koivumaa-Honkanen H, Honkanen R, Viinamäki H, Heikkilä K, Kaprio J, Koskenvuo M (2001). Life satisfaction and suicide: a 20-year follow-up study. Am J Psychiatry.

[18] Boehm JK, Kubzansky LD (2012). The heart's content: the association between positive psychological well-being and cardiovascular health. Psychol Bull.

[19] Okely JA, Cooper C, Gale CR (2016). Wellbeing and arthritis incidence: the survey of health, ageing and retirement in Europe. Ann Behav Med.

[20] Okely JA, Gale CR (2016). Well-being and chronic disease incidence: the English longitudinal study of ageing. Psychosom Med.

[21] Okely JA, Shaheen SO, Weiss A, Gale CR (2017). Wellbeing and chronic lung disease incidence: the survey of health, ageing and retirement in Europe. PLoS One.

[22] Kim ES, Kubzansky LD, Soo J, Boehm JK (2017). Maintaining healthy behavior: a prospective study of psychological well-being and physical activity. Ann Behav Med.

[23] Liu B, Floud S, Pirie K, Green J, Peto R, Beral V, Million Women Study Collaborators (2016). Does happiness itself directly affect mortality? The prospective UK million women study. Lancet.

[24] Diener E, Chan MY (2011). Happy people live longer: subjective well-being contributes to health and longevity. Appl Psychol Health Well Being.

[25] Carr A, Cullen K, Keeney C, Canning C, Mooney O, Chinseallaigh E, O’Dowd A (2020). Effectiveness of positive psychology interventions: a systematic review and meta-analysis. J Posit Psychol.

[26] van Agteren J, Iasiello M, Lo L, Bartholomaeus J, Kopsaftis Z, Carey M, Kyrios M (2021). A systematic review and meta-analysis of psychological interventions to improve mental wellbeing. Nat Hum Behav.

[27] Davis DE, Choe E, Meyers J, Wade N, Varjas K, Gifford A, Quinn A, Hook JN, Van Tongeren DR, Griffin BJ, Worthington EL (2016). Thankful for the little things: a meta-analysis of gratitude interventions. J Couns Psychol.

[28] Buchanan KE, Bardi A (2010). Acts of kindness and acts of novelty affect life satisfaction. J Soc Psychol.

[29] Regan A, Margolis S, Ozer DJ, Schwitzgebel E, Lyubomirsky S (2023). What is unique about kindness? Exploring the proximal experience of prosocial acts relative to other positive behaviors. Affect Sci.

[30] Gander F, Wagner L, Niemiec RM (2024). Do character strengths-based interventions change character strengths? Two randomized controlled intervention studies. Collabra Psychol.

[31] Carr A, Finneran L, Boyd C, Shirey C, Canning C, Stafford O, Lyons J, Cullen K, Prendergast C, Corbett C, Drumm C, Burke T (2023). The evidence-base for positive psychology interventions: a mega-analysis of meta-analyses. J Posit Psychol.

[32] Heintzelman SJ, Kushlev K, Lutes LD, Wirtz D, Kanippayoor JM, Leitner D, Oishi S, Diener E (2020). ENHANCE: evidence for the efficacy of a comprehensive intervention program to promote subjective well-being. J Exp Psychol Appl.

[33] Martin CC (2022). ENHANCE-II: an abridged intervention to promote subjective well-being. Int J Appl Posit Psychol.

[34] Prydz MB, Czajkowski NO, Eilertsen M, Røysamb E, Nes RB (2024). A web-based intervention using "five ways to wellbeing" to promote well-being and mental health: randomized controlled trial. JMIR Ment Health.

[35] Addington EL, Cummings P, Jackson K, Yang D, Moskowitz JT (2023). Exploring retention, usage, and efficacy of web-based delivery of positive emotion regulation skills during the COVID-19 pandemic. Affect Sci.

[36] Fleming T, Bavin L, Lucassen M, Stasiak K, Hopkins S, Merry S (2018). Beyond the trial: systematic review of real-world uptake and engagement with digital self-help interventions for depression, low mood, or anxiety. J Med Internet Res.

[37] Park Y, Guevarra DA, Simon-Thomas E, Epel ES (2024). Who engages in well-being interventions? An analysis of a global digital intervention study. J Posit Psychol.

[38] Beatty L, Binnion C (2016). A systematic review of predictors of, and reasons for, adherence to online psychological interventions. Int J Behav Med.

[39] Borghouts J, Eikey E, Mark G, De Leon C, Schueller SM, Schneider M, Stadnick N, Zheng K, Mukamel D, Sorkin DH (2021). Barriers to and facilitators of user engagement with digital mental health interventions: systematic review. J Med Internet Res.

[40] Riley RD, Lambert PC, Staessen JA, Wang J, Gueyffier F, Thijs L, Boutitie F (2008). Meta-analysis of continuous outcomes combining individual patient data and aggregate data. Stat Med.

[41] Stewart LA, Tierney JF (2002). To IPD or not to IPD? Advantages and disadvantages of systematic reviews using individual patient data. Eval Health Prof.

[42] Weiss LA, Westerhof GJ, Bohlmeijer ET (2016). Can we increase psychological well-being? The effects of interventions on psychological well-being: a meta-analysis of randomized controlled trials. PLoS One.

[43] Hendriks T, Warren MA, Schotanus-Dijkstra M, Hassankhan A, Graafsma T, Bohlmeijer E, de Jong J (2018). How WEIRD are positive psychology interventions? A bibliometric analysis of randomized controlled trials on the science of well-being. J Posit Psychol.

[44] Cheung F, Lucas RE (2014). Assessing the validity of single-item life satisfaction measures: results from three large samples. Qual Life Res.

[45] Gardner DG, Cummings LL, Dunham RB, Pierce JL (1998). Single-item versus multiple-item measurement scales: an empirical comparison. Educ Psychol Meas.

[46] Fredrickson BL, Tugade MM, Waugh CE, Larkin GR (2003). What good are positive emotions in crises? A prospective study of resilience and emotions following the terrorist attacks on the United States on September 11th, 2001. J Pers Soc Psychol.

[47] Shiota MN, Campos B, Oveis C, Hertenstein MJ, Simon-Thomas E, Keltner D (2017). Beyond happiness: building a science of discrete positive emotions. Am Psychol.

[48] Adler NE, Epel ES, Castellazzo G, Ickovics JR (2000). Relationship of subjective and objective social status with psychological and physiological functioning: preliminary data in healthy, White women. Health Psychol.

[49] Singh-Manoux A, Adler NE, Marmot MG (2003). Subjective social status: its determinants and its association with measures of ill-health in the Whitehall II study. Soc Sci Med.

[50] Benjamini Y, Hochberg Y (1995). Controlling the false discovery rate: a practical and powerful approach to multiple testing. J R Stat Soc Series B Stat Methodol.

[51] Cohen S, Williamson GM (1991). Stress and infectious disease in humans. Psychol Bull.

[52] Segerstrom SC, Miller GE (2004). Psychological stress and the human immune system: a meta-analytic study of 30 years of inquiry. Psychol Bull.

[53] Epel ES, Blackburn EH, Lin J, Dhabhar FS, Adler NE, Morrow JD, Cawthon RM (2004). Accelerated telomere shortening in response to life stress. Proc Natl Acad Sci U S A.

[54] Mathur MB, Epel ES, Kind S, Desai M, Parks CG, Sandler DP, Khazeni N (2016). Perceived stress and telomere length: a systematic review, meta-analysis, and methodologic considerations for advancing the field. Brain Behav Immun.

[55] Schutte NS, Malouff JM (2016). The relationship between perceived stress and telomere length: a meta-analysis. Stress Health.

[56] Kivimäki M, Steptoe A (2018). Effects of stress on the development and progression of cardiovascular disease. Nat Rev Cardiol.

[57] Cohen S, Janicki-Deverts D, Miller GE (2007). Psychological stress and disease. JAMA.

[58] Lorem G, Cook S, Leon D, Emaus N, Schirmer H (2020). Self-reported health as a predictor of mortality: a cohort study of its relation to other health measurements and observation time. Sci Rep.

[59] Wu S, Wang R, Zhao Y, Ma X, Wu M, Yan X, He J (2013). The relationship between self-rated health and objective health status: a population-based study. BMC Public Health.

[60] Idler EL, Benyamini Y (1997). Self-rated health and mortality: a review of twenty-seven community studies. J Health Soc Behav.

[61] Jylhä M (2009). What is self-rated health and why does it predict mortality? Towards a unified conceptual model. Soc Sci Med.

[62] Reinwarth AC, Wicke FS, Hettich N, Ernst M, Otten D, Brähler E, Wild PS, Münzel T, König J, Lackner KJ, Pfeiffer N, Beutel ME (2023). Self-rated physical health predicts mortality in aging persons beyond objective health risks. Sci Rep.

[63] Sella E, Miola L, Toffalini E, Borella E (2023). The relationship between sleep quality and quality of life in aging: a systematic review and meta-analysis. Health Psychol Rev.

[64] Kwok CS, Kontopantelis E, Kuligowski G, Gray M, Muhyaldeen A, Gale CP, Peat GM, Cleator J, Chew-Graham C, Loke YK, Mamas MA (2018). Self-reported sleep duration and quality and cardiovascular disease and mortality: a dose-response meta-analysis. J Am Heart Assoc.

[65] Götz FM, Gosling SD, Rentfrow PJ (2022). Small effects: the indispensable foundation for a cumulative psychological science. Perspect Psychol Sci.

[66] Rosenthal R, DiMatteo MR (2001). Meta-analysis: recent developments in quantitative methods for literature reviews. Annu Rev Psychol.

[67] Rosnow RL, Rosenthal R (2003). Effect sizes for experimenting psychologists. Can J Exp Psychol.

[68] Cohen J (1988). Statistical Power Analysis for the Behavioral Sciences. 2nd edition.

[69] Morozink JA, Friedman EM, Coe CL, Ryff CD (2010). Socioeconomic and psychosocial predictors of interleukin-6 in the MIDUS national sample. Health Psychol.

[70] Eid M, Diener E (2004). Global judgments of subjective well-being: situational variability and long-term stability. Soc Indic Res.

[71] Hudson NW, Lucas RE, Donnellan MB (2017). Day-to-day affect is surprisingly stable: a two-year longitudinal study of well-being. Soc Psychol Personal Sci.

[72] Newman DB (2022). Low income amplifies the negative relationship between nostalgia proneness and well-being. Appl Res Qual Life.

