# Supplementary material for: Scaling a Brief Digital Well-Being Intervention (the Big Joy Project) and Sociodemographic Moderators: Single-Group Pre-Post Study
Source: J Med Internet Res. 2025 Jun 4;27:e72053. doi: 10.2196/72053 (PMC12177429; doi:10.2196/72053)
Supplement: Multimedia Appendix 1 [file jmir_v27i1e72053_app1.docx]

| Multimedia Appendix 1: Moderation by dose (continuous) | | | |
| --- | --- | --- | --- |
| Outcomes | *β (SE)* | *95% CI* | *P* |
| **Emotional well-being** |  |  |  |
| Mean – 1 SD | 0.87 (0.02) | (0.83 to 0.90) | <.001 |
| Mean + 1SD | 1.23 (0.02) | (1.19 to 1.26) | <.001 |
| **Positive emotions** |  |  |  |
| Mean – 1 SD | 0.75 (0.02) | (0.72 to 0.79) | <.001 |
| Mean + 1SD | 1.15 (0.02) | (1.11 to 1.19) | <.001 |
| **Happiness agency** |  |  |  |
| Mean – 1 SD | 0.74 (0.02) | (0.69 to 0.78) | <.001 |
| Mean + 1SD | 1.21 (0.02) | (1.16 to 1.25) | <.001 |
| **Perceived stress** |  |  |  |
| Mean – 1 SD | -0.82 (0.03) | (-0.88 to -0.76) | <.001 |
| Mean + 1SD | -1.10 (0.03) | (-1.16 to -1.05) | <.001 |
| **Self-reported health** |  |  |  |
| Mean – 1 SD | 0.05 (0.01) | (0.04 to 0.06) | <.001 |
| Mean + 1SD | 0.09 (0.01) | (0.08 to 0.11) | <.001 |
| **Sleep quality** |  |  |  |
| Mean – 1 SD | 0.13 (.01) | (0.12 to 0.15) | <.001 |
| Mean + 1SD | 0.16 (.01) | (0.15 to 0.18) | <.001 |
| Note: This table presents simple slopes of outcomes at low (Mean – 1 SD) and high (Mean + 1 SD) levels of dose, modeled as a continuous moderator. All estimates are unstandardized regression coefficients (*β*) with standard errors (*SE*) in parentheses. The 95% confidence intervals (*CI*) and associated *P* values indicate the strength and precision of each effect. Across all outcomes, higher dose levels were associated with more favorable outcomes, including greater emotional well-being, positive emotions, happiness agency, and self-reported health, better sleep quality, well as lower perceived stress. All effects were statistically significant at *P*<.001. | | | |
