# Supplementary material for: Scaling a Brief Digital Well-Being Intervention (the Big Joy Project) and Sociodemographic Moderators: Single-Group Pre-Post Study
Source: J Med Internet Res. 2025 Jun 4;27:e72053. doi: 10.2196/72053 (PMC12177429; doi:10.2196/72053)
Supplement: Multimedia Appendix 2 [file jmir_v27i1e72053_app2.docx]

| Multimedia Appendix 2: Moderation by dose (factor) | | | | | | | | | | | | | |  |
| --- | --- | --- | --- | --- | --- | --- | --- | --- | --- | --- | --- | --- | --- | --- |
|  |  | Emotional  well-being | | Positive  emotions | | Happiness  agency | | Perceived  stress | | Self-reported  health | | Sleep  quality | | |
| Dose | *n* | *d_z_* | *P* vs. dose 0 | *d_z_* | *P* vs. dose 0 | *d_z_* | *P* vs. dose 0 | *d_z_* | *P* vs. dose 0 | *d_z_* | *P* vs. dose 0 | *d_z_* | *P* vs. dose 0 | |
| 0 | 385 | .19^***^ | – | .13^**^ | – | .11^*^ | – | -.23^***^ | – | -.01 | – | .15^***^ | – | |
| 1 | 639 | .27^***^ | .034 | .19^***^ | .127 | .16^***^ | .338 | -.26^***^ | .820 | .01 | .509 | .09^**^ | .851 | |
| 2 | 912 | .36^***^ | .001 | .30^***^ | .003 | .25^***^ | .012 | -.27^***^ | .910 | .04 | .086 | .13^***^ | .851 | |
| 3 | 1,351 | .42^***^ | <.001 | .38^***^ | <.001 | .34^***^ | <.001 | -.31^***^ | .416 | .07^***^ | .017 | .13^***^ | .851 | |
| 4 | 1,931 | .42^***^ | <.001 | .40^***^ | <.001 | .35^***^ | <.001 | -.32^***^ | .413 | .06^***^ | .033 | .13^***^ | .851 | |
| 5 | 2,830 | .47^***^ | <.001 | .43^***^ | <.001 | .44^***^ | <.001 | -.32^***^ | .337 | .07^***^ | .010 | .13^***^ | .851 | |
| 6 | 4,171 | .53^***^ | <.001 | .51^***^ | <.001 | .50^***^ | <.001 | -.39^***^ | .022 | .08^***^ | .005 | .16^***^ | .880 | |
| 7 | 5,379 | .57^***^ | <.001 | .53^***^ | <.001 | .55^***^ | <.001 | -.41^***^ | .013 | .10^***^ | .001 | .17^***^ | .879 | |
| Note. We also treated dosage as a factorial variable, used an F test for significant interactions, and used a Wilcoxon Rank Sum Test to compare the pre-post change score with 0 dosages as a reference point. We used the Benjamini-Hochberg procedure for all post hoc analyses, which involves comparing each p-value to a critical value or threshold. We report adjusted p-values in the tables. There were *n*=385 to 407 (sample size depends on outcome variable) people who did not engage in any daily activities but completed the pre- and post-intervention surveys. We calculated effect sizes using within-subjects Cohen *d_z_*. ^*^*P*<.05, ^**^*P*<.01, ^***^*P*<.001 represents *P*-values for the pre-post at each dose. Post-hoc analysis compared dose 0 with doses 1 through 7. *P* vs. dose 0 represents adjusted *P*-values comparing dose 0 as a reference using Benjamini-Hochberg corrections. Sample size is based on emotional well-being sample. | | | | | | | | | | | | | |  |
