# Supplementary material for: Scaling a Brief Digital Well-Being Intervention (the Big Joy Project) and Sociodemographic Moderators: Single-Group Pre-Post Study
Source: J Med Internet Res. 2025 Jun 4;27:e72053. doi: 10.2196/72053 (PMC12177429; doi:10.2196/72053)
Supplement: Multimedia Appendix 3 [file jmir_v27i1e72053_app3.docx]

| Multimedia Appendix 3: Intercept and pre-post slope correlations | |
| --- | --- |
| Outcomes | Intercept – Slope Correlation |
| Emotional well-being | -.42 |
| Positive emotions | -.43 |
| Happiness agency | -.48 |
| Perceived stress | -.51 |
| Self-reported health | -.32 |
| Sleep quality | -.35 |
| Note: These correlations represent the intercept and pre-post slope correlations for the primary intervention analysis with dose as a continuous moderator. The correlations were similar for the other moderators. Negative correlations between random intercepts and pre-post slopes indicate that individuals with lower baseline values (higher for stress) showed greater improvements. | |
